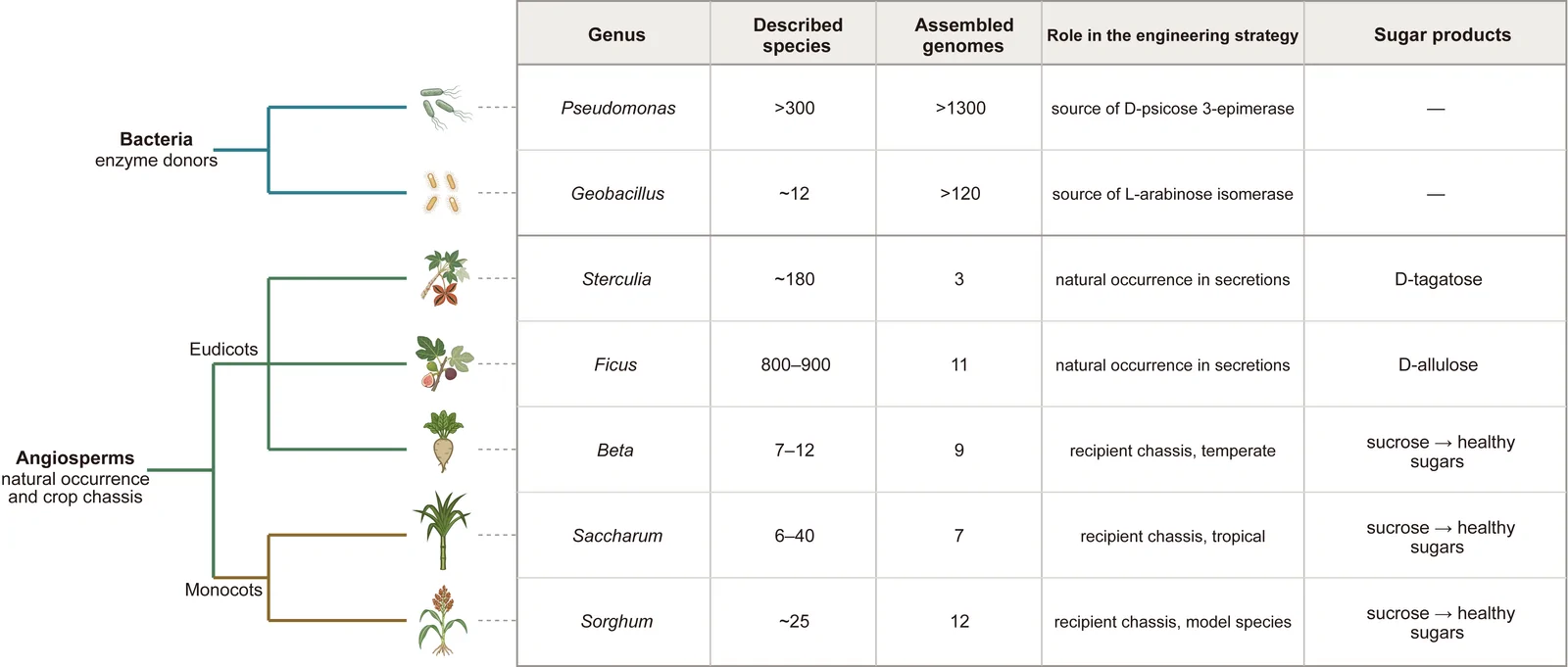# Sugar 1.0 to 4.0: From conventional sugar production to precision biomanufacturing of healthy sugars

**DOI:** 10.1016/j.bidere.2026.100125

**Published:** 2026-10-01

**Authors:** Zihao Zhang, Junlin Li, Jiahao Zhang, Qibin Wu, Fei Chen, Youxiong Que

**Affiliations:** a State Key Laboratory of Tropical Crop Breeding, Institute of Tropical Bioscience and Biotechnology, Sanya Research Institute, Chinese Academy of Tropical Agricultural Sciences, Sanya, Hainan, 572024, China; b State Key Laboratory of Tropical Crop Breeding, Sanya Institute of Breeding and Multiplication, Hainan University, Sanya, 572025, China

**Keywords:** Sugar 4.0, Healthy sugars, D-allulose, D-tagatose, Plant bio-factories

## Abstract

Excessive intake of free sugars is a major modifiable risk factor for obesity, type 2 diabetes and cardiovascular disease, and it is a global public-health priority to reduce dietary sugar while preserving sensory quality. Referred to here as “healthy sugars”, the low-calorie monosaccharides D-allulose and D-tagatose combine sucrose-like sensory and bulking behavior with a distinct metabolic fate in humans and, for D-tagatose, with defence-inducing activity in plants. This review traces the evolution of sugar supply through four stages, from agricultural extraction of sucrose (Sugar 1.0) and enzymatic hydrolysis to high-fructose syrup (Sugar 2.0) to chemically synthesized high-intensity sweeteners (Sugar 3.0) and precision biomanufacturing of healthy sugars (Sugar 4.0). We review the molecular basis of healthy-sugar action in mammalian metabolic homeostasis and in plant sweet immunity, then compare manufacturing platforms, equilibrium-limited enzymatic epimerization, immobilized-enzyme and continuous-reactor processes, thermodynamically driven cell-free cascades, and cell-free CO_2_-to-hexose synthesis, through a consolidated cross-platform comparison of conversion, titer, yield, scalability and technology readiness. We then set out an amyloplast-targeted compartmentalised design for sugar-crop chassis, a distinct phosphorylated route for D-tagatose, and a climate-adaptive sugarcane, sugar beet, sorghum manufacturing crops. Finally we examine the conditions under which a plant-chassis route could offer cost or carbon advantages, comparing feedstock, energy, separation and carbon-balance considerations across platforms, and comparing the ingredient and biosafety requirements applying in the United States, the European Union and China. The plant-chassis route is presented throughout as a design proposal requiring crop-level validation, and the prerequisites for its projected advantages are stated explicitly.

## 1 Introduction

Sucrose is primarily extracted from tropical sugarcane (*Saccharum* spp. hybrids, ∼80% of global production) and temperate sugar beet (*Beta vulgaris* subsp. *vulgaris*, ∼20% of global production). It has long been regarded as the “sensory gold standard” for sweeteners due to its pure sweetness profile and excellent processing characteristics, such as providing bulk, moisture retention, and participation in the Maillard reaction [1]. However, as the link between excessive sugar intake and global health crises such as obesity, diabetes, and cardiovascular diseases becomes increasingly clear, reducing sucrose has become an urgent public health need worldwide [2]. According to statistics, the current global annual consumption of sucrose has reached approximately 175 million tons, behind which lies a severe public health challenge [3]. The World Health Organization (WHO) has clearly defined “Free Sugars” and recommends limiting their intake to less than 10% of total energy intake, with a further reduction to 5% [2]. Yet epidemiological surveys show average free sugar intake accounts for 11% of total energy in adults and 12%-14% in children/adolescents [4], and the innate human preference for sweetness makes it extremely difficult to meet dietary standards by willpower alone.

To maintain sweetness while reducing sugar, the food industry has long relied on non-nutritive sweeteners (NNS). However, the sensory performance and metabolic effects of NNS vary considerably between compounds, doses and exposure patterns. Moreover, questions about long-term metabolic consequences remain unresolved, which sustains interest in alternative sugar-reduction strategies [5,6].

Human access to sweetness has evolved through three eras: Sugar 1.0 (agricultural extraction of sucrose) [7,8], Sugar 2.0 (enzymatic hydrolysis for high-fructose syrup) [9], and Sugar 3.0 (chemical synthesis of high-intensity sweeteners) [10]. All three generations fail to reconcile health benefits with sensory quality, driving the industry shift from “sugar reduction” to “sugar upgrading” and the advent of Sugar 4.0: precision biomanufacturing of healthy sugars (Fig. 1).

**Fig. 1:**
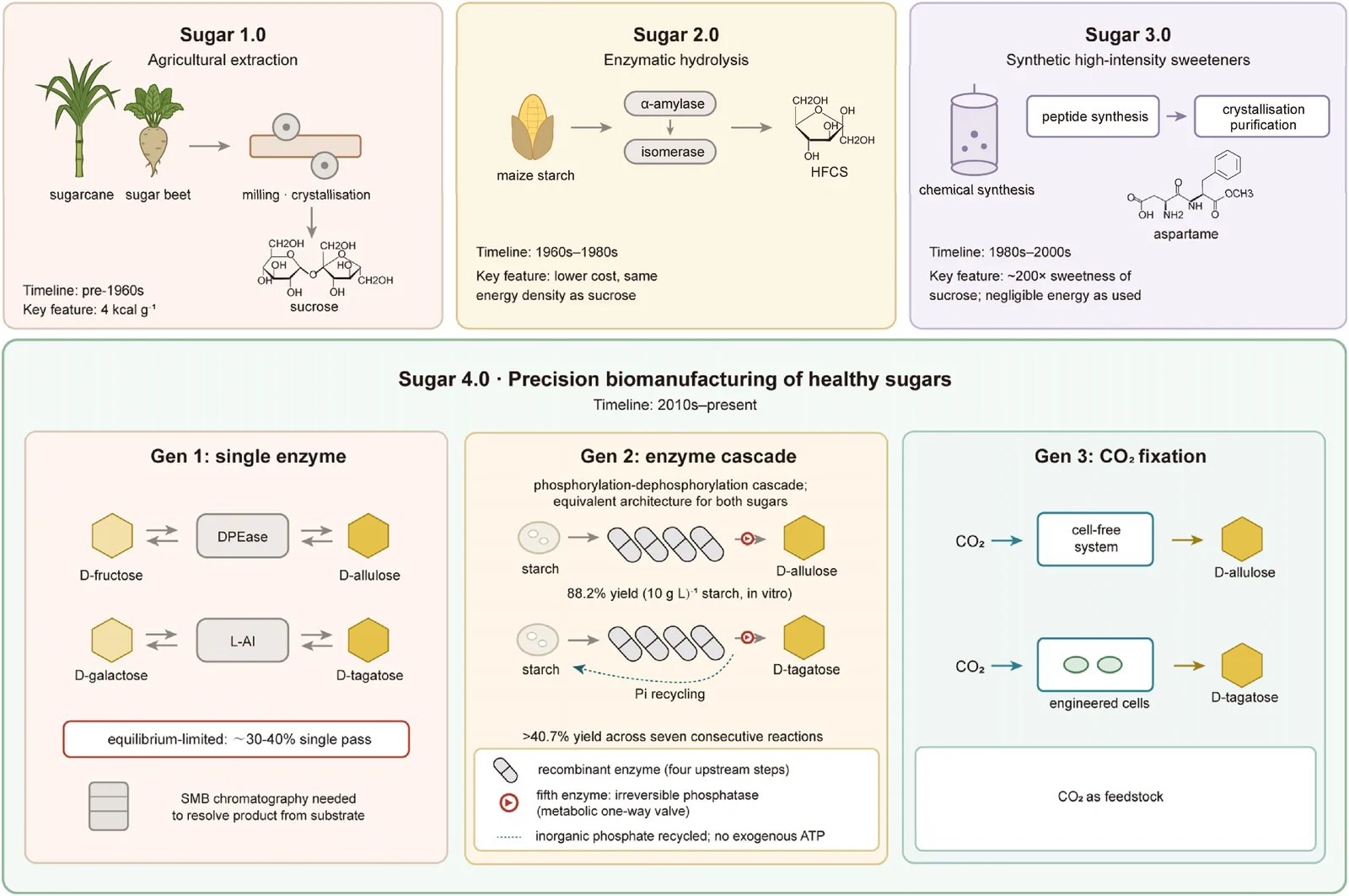
Four technological generations of sugar manufacturing. Sugar 1.0, agricultural extraction of sucrose; Sugar 2.0, enzymatic hydrolysis and isomerization of starch to high-fructose syrup; Sugar 3.0, chemical synthesis of high-intensity sweeteners; Sugar 4.0, precision biomanufacturing of the healthy sugars D-allulose and D-tagatose. Within Sugar 4.0, Gen 1 denotes single-enzyme epimerization or isomerization, constrained by the reaction equilibrium; Gen 2 denotes phosphorylation-dephosphorylation cascades in which the terminal dephosphorylation is thermodynamically irreversible and therefore acts as a metabolic one-way valve (one-way valve symbol), removing the equilibrium ceiling, with inorganic phosphate recycled in a closed loop and no exogenous ATP required (dashed line); Gen 3 denotes cell-free or whole-cell fixation of CO_2_. Yields shown are those reported in the cited studies (D-allulose, [11]; D-tagatose, [12]) and were not obtained under harmonized conditions. No comparative life-cycle assessment of these routes has been published. Gen, Generation. DPEase, D-psicose 3-epimerase.

The “healthy sugars” discussed here are the naturally occurring functional monosaccharides D-allulose and D-tagatose [13,14]. Sugar alcohols such as erythritol are excluded because of reported cardiovascular safety concerns [15], and isomaltulose is excluded as a fully digestible disaccharide providing approximately 4 kcal g^−1^ [16], which is inconsistent with the “metabolic escape” logic that defines the present scope. D-allulose and D-tagatose are naturally occurring ketohexoses: D-allulose is the C-3 epimer of D-fructose and D-tagatose is its C-4 epimer. D-tagatose is also the ketose isomer of D-galactose, which is the basis of the L-arabinose isomerase route described below. Both participate in the Maillard reaction with amino acids [17].

Most existing reviews on healthy sugars focus on single dimensions of enzymatic modification or microbial fermentation [18], lacking a systematic review integrating the full industrial chain from molecular physiological mechanisms, synthetic biology-driven manufacturing, to crop chassis engineering and climate-adaptive industrial layout. This review fills this gap by systematically sorting out the four technological iterations of the global sugar industry, elaborating the dual biological functions of healthy sugars in human metabolism and plant immunity. We propose to construct a climate-adaptive global manufacturing crops based on *Saccharum* spp. hybrids, *B. vulgaris* subsp. *vulgaris* and *S. bicolor*, and discuss industry challenges and prospects, to provide a comprehensive theoretical framework for the sugar industry's transition to precision biomanufacturing.

## 2 Important role of healthy sugars in human metabolism

Traditional sugar biology often regards sugars merely as energy carriers, while D-allulose and D-tagatose act as “non-metabolic signaling molecules” in both animal and plant cells, showing cross-species biological conservation. This chapter elucidates how this mechanism provides dual protection for human metabolic homeostasis and plant innate immunity (Fig. 2a–b).

**Fig. 2:**
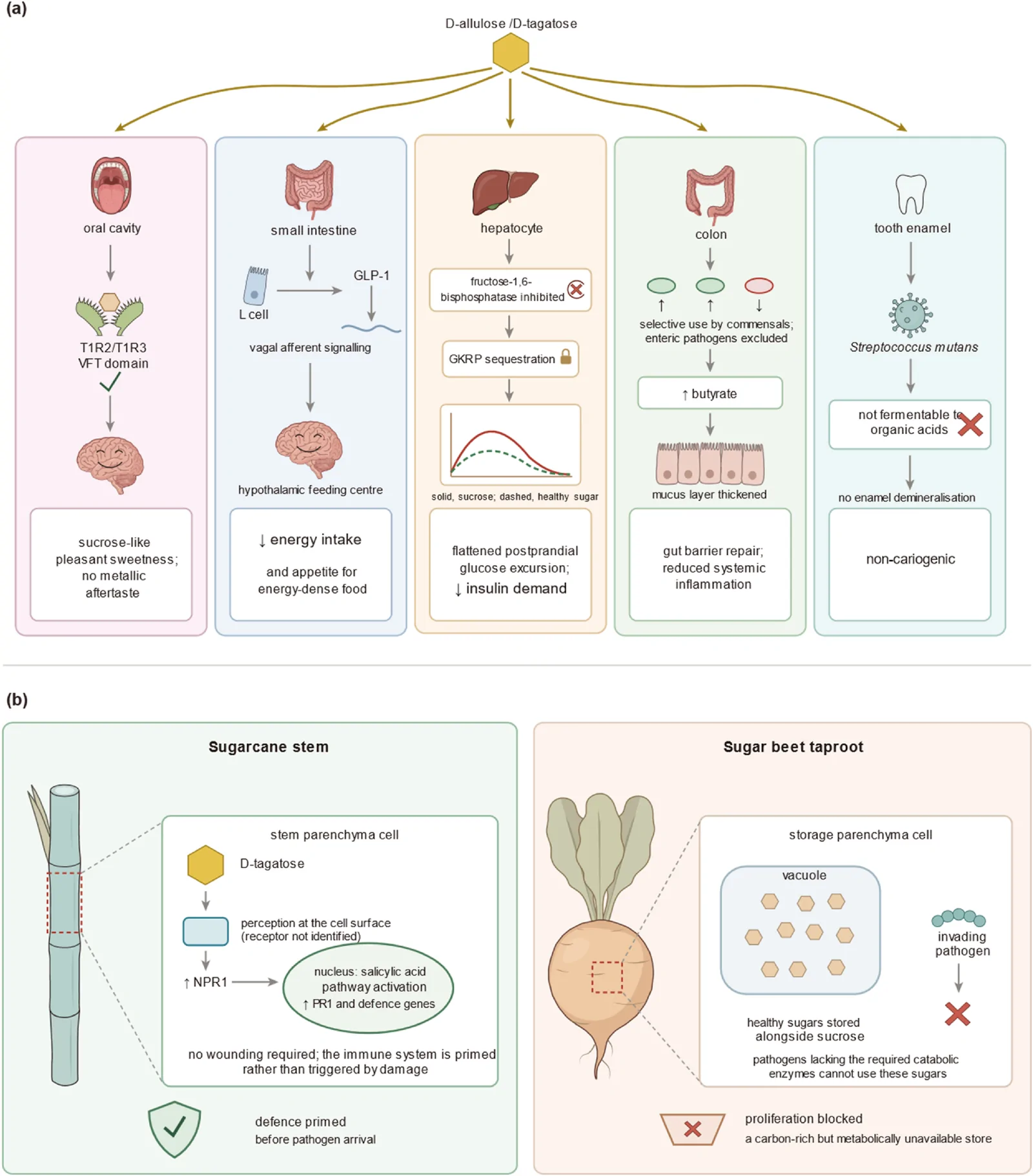
Mechanisms of healthy sugars in regulating mammalian metabolic homeostasis and plant innate immunity. (a) Regulatory pathways of healthy sugars in mammalian metabolism and oral health. (b) Sweet immunity mechanism of healthy sugars in plant disease resistance. The mechanisms shown are drawn from the plant-pathogen systems in which they have been reported. Their operation in sugarcane and sugar beet has not been established experimentally.

### 2.1 Metabolic remodeling and microecological optimization

Healthy sugars can replace sucrose as the “sensory gold standard” due to their precise binding to taste receptors. Molecular docking studies confirmed they can embed into the orthosteric site of the T1R2/T1R3 receptor's Venus flytrap domain as accurately as sucrose [19,20], activating nearly identical pleasure neural signals without metallic aftertaste (Fig. 2a). Their deeper value lies in active regulation of the “gut-brain axis”: D-allulose entering the small intestine induces intestinal L-cells to release glucagon-like peptide-1 (GLP-1), which signals to the central nervous system via the vagus nerve, alters hypothalamic feeding center activity, suppresses high-calorie food cravings, and shifts from passive dieting to active physiological management (Fig. 2a) [21].

Liver metabolism studies revealed healthy sugars’ blocking effect on the gluconeogenesis pathway. Fructose from sucrose hydrolysis is a potent gluconeogenic substrate that bypasses the rate-limiting enzyme phosphofructokinase to enter the metabolic pool, a key factor in insulin resistance [22]. In contrast, the two healthy sugars follow different hepatic routes. D-allulose is absorbed but largely excreted unchanged in the urine and contributes almost no carbon to hepatic glucose production, although a small fraction is phosphorylated by ketohexokinase to D-allulose 1-phosphate. D-tagatose is phosphorylated by hepatic fructokinase to D-tagatose 1-phosphate, which is cleaved only slowly, so a part of an ingested dose does enter glucose and glycogen synthesis. This partial metabolism is reflected in its energy factor of 1.5 kcal g^−1^, compared with 0.4 kcal g^−1^ for D-allulose [14,23]. D-allulose acts in addition through the glucokinase regulatory protein (GKRP): D-allulose 1-phosphate binds GKRP and releases glucokinase, then translocates from the nucleus to the cytoplasm and increases hepatic glucose phosphorylation and glycogen storage, flattening the postprandial glucose excursion (Fig. 2a) [23].

Unabsorbed healthy sugars reshape gut microecology via a “specificity screening” function. They selectively promote beneficial bacteria growth, are fermented by colonic microbiota to increase butyrate production, and repair the intestinal mucosal barrier [24,25], while pathogenic bacteria such as *Salmonella* spp. lacking corresponding metabolic enzymes are excluded, reducing systemic inflammation (Fig. 2a) [26].

Additionally, healthy sugars cannot be used as acid-producing substrates by cariogenic bacteria like *Streptococcus mutans*, fundamentally interrupting dental caries initiation (Fig. 2a) [27], with non-cariogenic properties that reinforce their population-wide dietary value.

### 2.2 Mechanism of sweet immunity in plants

The health attributes of healthy sugars transcend species, endowing plants with disease resistance. Unlike chemical pesticides’ lethal logic, healthy sugars induce endogenous plant defense via signal mimicry and nutritional competition regulation. D-tagatose treatment activates plant defence responses without physical damage, inducing the salicylic acid (SA) signalling pathway and upregulating *NPR1* and downstream defence genes such as *PR1* (Fig. 2b) [28]. Whether this response is initiated by a dedicated plasma-membrane receptor, as for canonical PAMPs, has not been established. The protection reported to date is not uniform across pathogens: in grapevine, D-tagatose reduced downy mildew but did not protect against gray mould (*Botrytis cinerea*), which is consistent with an SA-dependent response effective against biotrophs rather than necrotrophs.

A second, nutritional component has been proposed. Pathogens that lack the corresponding catabolic enzymes cannot use D-allulose or D-tagatose as a carbon source, so tissue in which these sugars accumulate alongside sucrose would present a carbon-rich but partly unavailable substrate to such organisms (Fig. 2b) [29]. This effect would apply only where the pathogen gains access to the stored pool, for example after tissue damage or during necrotrophic colonisation, since the sugars are sequestered in the vacuole. Its scope across pathogen groups has not been determined.

## 3 Production of healthy sugar

Healthy sugar biomanufacturing began with natural isomerase exploration. The “Izumoring” strategy proposed by Professor Izumori established the foundation for healthy sugar production by enzymatically linking all six-carbon sugar structural maps [30], with enzyme-catalyzed disaccharide rearrangement technologies forming the enzymatic basis for first-generation healthy sugar biomanufacturing [31].

### 3.1 Monosaccharide isomerization

First-generation production relies on single-step enzymatic isomerization: DPEase/DAEase catalyze D-fructose to D-allulose, and L-AI converts D-galactose to D-tagatose [[31], [32], [33]]. However, these reversible reactions are limited by thermodynamic equilibrium, with single-pass conversion typically about 30% and not exceeding roughly 40% even at elevated temperature, requiring costly SMB chromatography for purification (Fig. 1) [31,34].

Because single-pass conversion is capped by the epimerization equilibrium, the economics of first-generation processes are governed less by intrinsic catalytic activity than by catalyst lifetime, reactor productivity and separation duty. Three complementary lines of process intensification have therefore been pursued: enzyme immobilization, reactor engineering, and cascade catalysis.

**Enzyme immobilization and stabilization.** Immobilization converts a soluble epimerase into a recoverable, reusable catalyst and generally raises its thermal tolerance. D-psicose 3-epimerase immobilized on graphene oxide showed improved thermal stability and bioconversion efficiency [35]; encapsulation in magnetic metal-organic-framework nanoparticles allowed magnetic recovery and repeated reuse [36]; and immobilization on functionalised polyhydroxyalkanoate nano-beads produced a stable biocatalyst designed for cost-effective D-allulose production [37]. These formats are complementary to protein engineering: rational introduction of new disulfide bridges and proline substitution have each raised the thermostability of *Clostridium bolteae* D-allulose 3-epimerase [38,39], a precondition for operating at the elevated temperatures that shift the epimerization equilibrium towards the ketose product and suppress microbial contamination during long runs.

**Reactor engineering.** Immobilized D-tagatose 3-epimerase has been operated in a continuous bioreactor for mass production of D-allulose from D-fructose [40], and packed-bed and membrane configurations retain the catalyst while product is withdrawn continuously. A recent packed-bed system shows what sustained operation now looks like: a thermostable D-allulose 3-epimerase immobilized on a chitosan-diatomaceous-earth composite ran continuously for 150 days and delivered 756 kg of D-allulose, corresponding to 89.7% of the theoretical maximum for that feed, while the single-pass conversion declined only from 26.0% to 22.5% [41]. The two figures describe different quantities: the cumulative output is large because the reactor operated for five months, whereas each pass remains bounded by the epimerization equilibrium. Continuous operation matters beyond throughput: it permits in-line coupling to product removal, which displaces the equilibrium and raises effective conversion above the batch ceiling, and it exposes the true operating constraints of an immobilized system, enzyme half-life, pressure drop, carrier attrition and support replacement cost. Reported catalyst-lifetime data remain the most useful single indicator of whether a laboratory immobilization strategy will survive scale-up.

**Cascade catalysis.** The third strategy removes the equilibrium constraint rather than working around it, by coupling epimerization to an irreversible step within a multi-enzyme cascade. Because these designs also change the feedstock, from purified fructose or galactose to starch or maltodextrin, they are treated separately below. Their principal economic effect is indirect: higher single-pass yield reduces the load on the simulated moving-bed chromatography that remains the reference separation for D-allulose-D-fructose mixtures and a dominant contributor to unit cost [42].

### 3.2 Rare sugars as a molecular and technological resource pool

D-allulose and D-tagatose are the two most technologically advanced members of a much larger family of rare sugars. The Izumoring framework connects all hexoses through a limited set of isomerase, epimerase and reductase reactions, so that an enzyme or process innovation developed for one molecule is frequently transferable to others [30,43,44]. Two distinct contributions therefore flow from the wider rare sugar field into the healthy sugar programme: additional candidate molecules with untested sensory and metabolic profiles, and a shared toolkit of enzymes, immobilization chemistries and cascade architectures. Table 1 summarises representative rare sugars, their reported importance, and the most significant recent advances in their synthesis.

**Table 1:** Representative rare sugars, their reported importance, and recent advances in their biocatalytic synthesis.

Rare sugar	Reported importance	Representative advance in synthesis	Quantitative performance reported	References
D-allulose (D-psicose)	Ketohexose epimer of D-fructose; sucrose-like sweetness and bulking; energy factor of 0.4 kcal g^−1^ accepted by US FDA for labeling; attenuates postprandial glycaemic response	Thermodynamically driven *in vitro* cascade from starch; immobilized and engineered D-allulose 3-epimerase, including long-term continuous packed-bed operation; whole-cell routes from glucose or sucrose	88.2% yield from 10 g L^−1^ starch or maltodextrin and 79.2% from 50 g L^−1^ starch (217.8 mM); free-enzyme epimerization limited to ∼30-40% single-pass conversion; 756 kg produced over 150 d of continuous packed-bed operation, with single-pass conversion declining from 26.0% to 22.5%	[11,31,37,41,45]
D-tagatose	Ketohexose; C-4 epimer of D-fructose and ketose isomer of D-galactose; sweetness close to that of sucrose with an energy factor of 1.5 kcal g^−1^ accepted by US FDA; low glycaemic response; colonic fermentation to butyrate; elicits plant defence responses	Semiartificial cell factory converting starch through a phosphorylation-dephosphorylation cascade; whole-cell multienzyme cascade from D-fructose	>50% of initial activity retained across 20 consecutive batches spanning 480 h and >40.7% yield across seven consecutive reactions; 22.6 g L^−1^ and 75% conversion from 30 g L^−1^ D-fructose, rising to 68.1 g L^−1^ in fed-batch	[12,24,28,46]
D-allose	Aldohexose accessible within the Izumoring network; bioactivities under investigation; no established bulk food application	Enzymatic interconversion from D-psicose using ribose-5-phosphate isomerase and related enzymes	Laboratory-scale bioproduction; no consolidated process metrics	[30,44]
Other rare hexoses and pentoses (e.g. D-sorbose, L-sugars)	Structural diversity that defines the discovery space for future healthy sugars; physiological data largely absent	Enzyme mining, protein engineering and cascade design within the Izumoring framework	Heterogeneous laboratory evidence; metrics not comparable across studies	[31,43,44]

Note: Entries summarise properties and synthetic performance as reported in the cited studies. The values were obtained under different conditions and are not directly comparable across rows.

### 3.3 Synthetic biology-driven manufacturing

To overcome this equilibrium ceiling, synthetic biology introduced systems engineering design concepts, initiating a second wave of healthy sugar manufacturing. Researchers designed artificial metabolic pathways with a “Metabolic Ratchet” mechanism coupled with high-energy bond hydrolysis, forcing the reaction toward the product side and fundamentally overcoming chemical equilibrium limitations [47].

For D-allulose production, a phosphorylation-dephosphorylation cascade strategy was designed in which the final dephosphorylation step is irreversible, so that the theoretical conversion approaches completion and the pathway functions as a metabolic one-way valve (Fig. 1) [47]. The pathway comprises three sequential reactions: a kinase phosphorylates D-fructose to D-fructose 6-phosphate (F6P), an epimerase converts F6P to D-allulose 6-phosphate (A6P), and a specific phosphatase dephosphorylates A6P to release D-allulose. Implemented *in vitro* from starch or maltodextrin with α-glucan phosphorylase, phosphoglucomutase, phosphoglucose isomerase, A6P epimerase and A6P phosphatase, this architecture gave D-allulose yields of 88.2% from 10 g L^−1^ substrate and 79.2% from 50 g L^−1^ substrate, corresponding to 217.8 mM product [11]. The equivalent design for D-tagatose replaces costly fructose or galactose with starch or maltodextrin and couples the cascade to a closed-loop inorganic phosphate (Pi) cycle, which avoids exogenous ATP consumption [12]. Encapsulating the enzyme-bearing cells within an organosilica network allowed more than 50% of the initial activity to be retained across 20 consecutive batches spanning 480 h, with D-tagatose yields above 40.7% sustained over seven consecutive reactions [12]. A whole-cell multienzyme cascade operating from D-fructose through the same phosphorylated intermediate reached 75% conversion and 22.6 g L^−1^ D-tagatose from 30 g L^−1^ substrate, and 68.1 g L^−1^ under a fed-batch regime [46]. These figures define the performance that has actually been demonstrated for each architecture.

### 3.4 Cross-platform comparison of manufacturing performance

Reported performance metrics are not directly comparable unless conversion, titer and yield are stated separately, the feedstock is specified, and the demonstrated scale is made explicit. A high molar yield obtained at 10 g L^−1^ substrate in a laboratory reaction is not equivalent to a lower yield sustained in a continuous industrial reactor. Table 2 therefore consolidates the publicly available data for D-allulose and D-tagatose across the principal manufacturing platforms, reporting each metric only where the cited source provides it, and adding the principal cost driver and an author-assigned technology readiness level (TRL). Unit costs are recorded as not reported (NR) wherever no peer-reviewed, comparable figure exists. Commercial quotations have been excluded because they are region-specific, vary with contract volume and cannot be verified against a common basis. Two conclusions follow directly from the table. First, the platforms that have achieved industrial scale are precisely those constrained by the epimerization equilibrium, so their cost structure is dominated by separation rather than by conversion. Second, every platform that overcomes that equilibrium is currently at laboratory scale, and the plant-chassis route proposed in this review has no crop-level demonstration at all.

**Table 2:** Cross-platform comparison of reported manufacturing performance for D-allulose and D-tagatose.

Platform and feedstock	Conversion	Titer	Yield	Unit cost (US $∗kg^−1^) and principal cost driver	Scale demonstrated	TRL	References
D-allulose — free-enzyme epimerization (DAEase/DPEase); feedstock D-fructose	∼30-40% single pass (equilibrium-limited)	Process-dependent; not comparably reported	Equal to conversion	NR; SMB chromatographic separation of D-allulose from residual D-fructose	Commercial production	9	[[31], [32], [33],^42^]
D-allulose — immobilized epimerase in a continuous reactor; feedstock D-fructose	Equilibrium-limited unless coupled to product removal; 26.0% falling to 22.5% single pass over 150 d of continuous operation	NR	NR	NR; enzyme half-life and carrier replacement	Continuous packed-bed reactor; 756 kg over 150 d (89.7% of theoretical maximum)	6-7	[[35], [36], [37],^40^,^41^]
D-allulose — *in vitro* thermodynamically driven cascade; feedstock starch or maltodextrin	Not reported separately from yield	217.8 mM (≈39 g L^−1^) from 50 g L^−1^ substrate	88.2% (10 g L^−1^); 79.2% (50 g L^−1^)	NR; supply and replacement of five recombinant enzymes, Pi recycling	Laboratory	3-4	[11]
D-allulose — engineered whole-cell *Escherichia coli*; feedstock D-glucose or sucrose	Up to 84.8% on D-glucose (fed-batch)	15.80 g L^−1^ (fed-batch, D-glucose); 11.15 g L^−1^ (5 L fermenter, sucrose)	0.62 g g^−1^ D-glucose; 0.208 g g^−1^ sucrose	NR; low titer, cell separation and downstream recovery	5 L fermenter	4	[[47], [48], [49]]
D-allulose — plant chassis (proposed in this review); feedstock photosynthate	Not determined	Not determined	Not determined	Not determined; agronomic yield penalty and separation from co-accumulated sucrose, glucose and fructose	No crop-level demonstration	1-2	This review
D-tagatose — L-arabinose isomerase; feedstock D-galactose or lactose hydrolysate	Equilibrium-limited	NR	NR	NR; galactose feedstock and downstream separation	Commercial production	8-9	[27,31]
D-tagatose — semiartificial cell factory, phosphorylation cascade; feedstock starch or maltodextrin	Not reported separately	NR	>40.7% across seven consecutive reactions	NR; biocatalyst lifetime (>50% activity retained across 20 consecutive batches spanning 480 h)	Laboratory, repeated batch	4	[12]
D-tagatose — whole-cell multienzyme cascade; feedstock D-fructose	75%	22.6 g L^−1^ (batch); 68.1 g L^−1^ (fed-batch)	Not reported separately	NR; polyphosphate/ATP regeneration and product recovery	Laboratory fed-batch	3-4	[46]
D-tagatose — plant chassis (proposed in this review); feedstock photosynthate	Not determined	Not determined	Not determined	Not determined; precursor partitioning and separation burden	No crop-level demonstration	1-2	This review

Note: NR, not reported in a peer-reviewed source under conditions comparable with the other entries. Commercial quotations are excluded as unverifiable against a common basis. Values are taken from the cited studies and were not obtained under harmonized conditions. TRL values are assigned by the present authors from the highest publicly documented scale of demonstration and are not regulatory or certified designations (Scale from low to high: 1-9).

### 3.5 From carbon dioxide to sugar: Cell-free fixation routes

The profound transformation of healthy sugar manufacturing is reflected in the gradual decoupling of production logic from traditional crop primary metabolism, with the core being synthetic biology's pathway reprogramming to directly use CO_2_ for industrial synthesis, breaking the natural abundance ceiling of healthy sugar synthesis.

A milestone study by Yang et al. achieved precise total synthesis of hexoses from CO_2_ in the laboratory, with a shorter, more energy-efficient artificial pathway than the Calvin Cycle, and a carbon conversion rate significantly higher than natural photosynthesis, providing a feasible “air-to-sugar” roadmap (Fig. 1) [50]. Cai et al.’s previously reported Artificial Starch Anabolic Pathway (ASAP) system, which achieves *de novo* CO_2_-to-starch synthesis, provides a suitable upstream interface for healthy sugar production [51].

In principle the ASAP system could be coupled to the starch-based phosphorylation-dephosphorylation cascade described above [12] to give a complete CO_2_-to-D-tagatose route. Such a coupling has not been demonstrated, and the two systems have not been operated under a common set of conditions.

These third-generation systems are all based on cell-free synthetic biology, which eliminates the carbon and energy dissipation associated with cellular growth and concentrates resources on product synthesis. It should be emphasized that removing growth-associated losses does not by itself establish a lower overall energy or carbon burden, the demand shifts to enzyme production, cofactor and polyphosphate supply, catalyst replacement and downstream separation, and no harmonized life-cycle assessment has yet compared these routes with agricultural production on a common functional unit. On present evidence the energy consumption and catalyst costs of artificial systems cannot support million-tonne bulk sugar production in the near term, which motivates a re-evaluation of plants as solar-powered CO_2_ capture and conversion systems. Transferring synthetic-biology design concepts from *in vitro* reactors into crop cells may combine precise product design with the low marginal cost of agricultural cultivation, provided that the conditions set out in the following sections are met.

## 4 Roadmap for healthy sugar biomanufacturing

Despite rapid advances in microbial fermentation and cell-free systems, plants retain a structural advantage for bulk production at the million-tonne scale: they fix carbon using sunlight and require no sterile fermentation infrastructure [52]. Whether that advantage translates into a lower delivered cost per kilogram of purified product has not been established, as no comparative life-cycle assessment of these routes has been published. The evolution of sugar production technology shows a clear transition path from traditional agricultural extraction (Sugar 1.0) to synthetic biology manufacturing (Sugar 4.0), reflecting a shift from “resource dependency” to “precision sustainable manufacturing” [11].

### 4.1 From natural discovery to engineering reshaping

D-tagatose occurs naturally in the gum exudate of *Sterculia setigera* Delile [53], and D-allulose has been measured at trace levels in a range of plant-derived foods, including wheat, raisins and dried figs from *Ficus carica* L [54]. (Supplementary Fig. S1). No dedicated rare-sugar biosynthetic gene cluster has been described in higher plants, and a proportion of the D-allulose measured in processed foods is formed from D-fructose by non-enzymatic rearrangement during heating rather than by the plant itself. The remainder is most plausibly a by-product of the substrate promiscuity of basal sugar-metabolizing enzymes. The *Sterculia exudate* is the clearer case. It shows that a higher plant can accumulate a rare ketohexose in living tissue without evident metabolic toxicity, although at concentrations far below those a production chassis would need to reach. The core high-efficiency biosynthetic tools (DPEase, L-AI) are forged by the microbial world through long-term competitive evolution, such as *Pseudomonas cichorii* and various extremophilic thermophiles (Supplementary Fig. S1) [55]. To achieve large-scale industrial supply, one rational strategy is to transplant these high-efficiency microbial enzyme genes into domesticated *Saccharum* spp. hybrids with high biomass and photosynthetic efficiency, reconstructing the efficient Sugar 4.0 biosynthetic network on an optimal agricultural chassis [56].

We developed a “Climate-Dependent” strategy integrating *Saccharum* spp. hybrids, *B. vulgaris* subsp. *vulgaris*, and *S. bicolor* into a complementary global biomanufacturing crops. As a tropical C4 crop, *Saccharum* spp. hybrids are among the most efficient terrestrial crops in converting solar energy and produce very large amounts of biomass, with mature stem vacuoles as natural sugar reservoirs, suitable for large-scale raw material accumulation [56,57]. However, its highly polyploid/aneuploid genome (8x-12x, ∼10 Gb) exponentially increases the difficulty of CRISPR/Cas9 multi-locus knockout, though its genetic transformation systems are relatively mature [58,59]. In temperate regions, diploid *B. vulgaris* subsp. *vulgaris* with a simple, high-quality sequenced genome is an ideal chassis for precise genome editing [60,61], with cold tolerance that forms a geographical complement to *Saccharum* spp. hybrids [62]. *S. bicolor*, a C4 close relative of *Saccharum* spp. with a simple diploid genome (∼730 Mb) and high collinearity with sugarcane [63], serves as a “functional model crop”. Its three-to four-month growth cycle allows up to three design-build-test-learn cycles per year under controlled conditions, acting as a rapid iteration pilot platform for sugarcane genetic manipulation, with drought/salt-alkali resistance that allows cultivation on marginal lands [64,65].

### 4.2 Engineering strategies for cell factories

The sink organs of sugar crops (sugarcane stalks, sugar beet taproots, sweet sorghum stem pith) are the primary sites for photosynthate accumulation, with over 90% of cell volume occupied by a large central vacuole, and endogenous carbon flux terminating in sucrose synthesis and vacuolar storage [8,56,66]. A highly active cytoplasmic sucrose-hexose futile cycle results in an extremely limited free hexose pool, with endogenous hexokinase/fructokinase exerting strong competitive consumption of exogenous hexoses. These make the cytoplasm unsuitable for efficient heterologous sugar production [67,68].

Long-distance photosynthate transport in higher plants requires sucrose as the sole phloem carrier, and substantial intervention in sucrose synthesis/transport will cause severe growth impairment or lethality [69]. Therefore, all metabolic engineering is strictly confined to mature sink organs, fully retaining source organ sucrose synthesis and phloem transport functions, with an independent heterologous biosynthetic module constructed exclusively in sink cell amyloplasts to enable parallel D-allulose and sucrose accumulation (Fig. 3a–b).

**Fig. 3:**
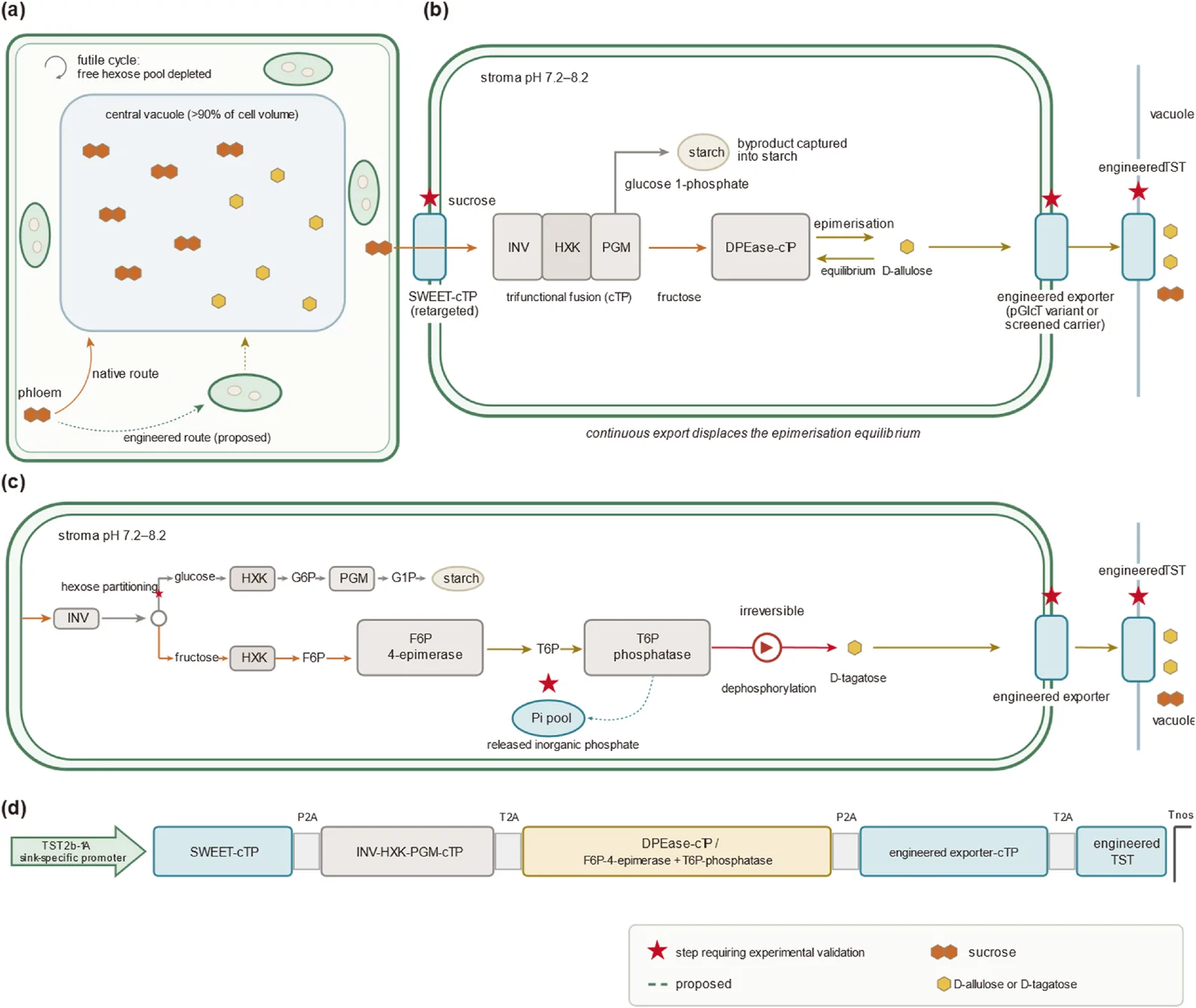
Proposed amyloplast-targeted compartmentalised system for healthy-sugar production in sugar-crop sink organs. (a) Whole storage parenchyma cell, showing the native sucrose storage route and the proposed engineered route operating in parallel. (b) Proposed catalytic cascade for D-allulose within the amyloplast stroma. (c) Proposed cascade for D-tagatose. The sucrose import and invertase steps are shared with (b), whereas hexose phosphorylation is route-specific: the fructose released by invertase is phosphorylated here, but must remain unphosphorylated in (b). The irreversible dephosphorylation removes the equilibrium ceiling that constrains isomerase-based routes. (d) Single-plasmid polycistronic expression cassette for all functional components, driven by a sink-specific promoter. Stars mark the steps identified in the text as requiring experimental validation: retargeting of SWEET to the amyloplast envelope, export across the envelope, vacuolar loading, partitioning of carbon between the engineered branch and starch synthesis, the substrate specificity of the hexose kinase in each route, and inorganic phosphate release into the stromal pool. The system is a design proposal and has not been demonstrated in any crop. Abbreviations: INV, invertase; HXK, hexokinase; PGM, phosphoglucomutase; cTP, chloroplast transit peptide used here for plastid targeting; F6P, D-fructose 6-phosphate; T6P, D-tagatose 6-phosphate; DPEase, D-psicose 3-epimerase; Pi, inorganic phosphate; P2A, porcine teschovirus-1 2A peptide; T2A, *Thosea asigna* virus 2A peptide; SWEET, Sugars Will Eventually be Exported Transporter; TST, tonoplast sugar transporter; pTST2b-1A, promoter of the sugarcane tonoplast sugar transporter allele TST2b-1A; pGlcT, plastidic glucose transporter; and Tnos, nopaline synthase terminator.

Amyloplasts in sink cells become the core reaction microcompartment for three key reasons: first, amyloplasts lack SPS and related hexose phosphate kinase components, eliminating futile cycle-related substrate competition [70]; second, the plastid double membrane provides a physical barrier isolating the heterologous pathway from the endogenous metabolic network [71]; third, the amyloplast stromal pH (7.2-8.2) is highly congruent with DPEase's optimal catalytic conditions, and the double membrane protects heterologous enzymes from cytosolic protease degradation [72,73].

We designed a single-plasmid polygenic amyloplast-targeted heterologous biosynthetic system for D-allulose production, integrating substrate supply, catalytic conversion, product export, and vacuolar storage modules into a single vector driven by a sink-specific promoter, with co-expression of all components from a single polycistronic transcript via P2A/T2A self-cleaving peptides and precise amyloplast targeting via cTP fusion (Fig. 3d) [74,75]. This single-plasmid strategy streamlines transformation and reduces the variability that arises when components are introduced as independent transgenes, although cleavage efficiency and differences in protein stability mean that equimolar accumulation of the mature proteins cannot be assumed.

The substrate supply module redirects a cTP-fused SWEET sucrose transporter to the amyloplast envelope, driving continuous sucrose influx via facilitated diffusion [74], with imported sucrose hydrolyzed by cTP-tagged neutral invertase. To prevent glucose leakage via the endogenous plastidic glucose transporter pGlcT [75], invertase, a glucose-specific hexokinase and phosphoglucomutase are fused into a trifunctional chimeric enzyme, so that the glucose released by invertase is phosphorylated and captured *in situ* and enters the endogenous starch pathway without metabolic toxicity [76]. The kinase must discriminate against D-fructose, because the free fructose pool is the substrate of the downstream epimerase. Plant hexokinases phosphorylate both hexoses, so selection or engineering of a glucose-specific enzyme is a further requirement to be tested. The retargeting of SWEET to the amyloplast envelope is the primary engineering bottleneck requiring experimental validation.

The catalytic module consists of cTP-tagged DPEase, which catalyzes fructose-to-D-allulose epimerization in the amyloplast stroma without ATP consumption. Continuous product export maintains low stromal D-allulose concentrations, perpetually displacing the epimerization equilibrium, with conversion potentially far exceeding the equilibrium ceiling of free-enzyme systems (about 30-40% single pass). The engineering foundation for DPEase is well-established, with extensive thermostability enhancement strategies documented in the literature [38,39].

For product export and storage, two parallel strategies are proposed for amyloplast envelope export: rational modification of pGlcT to broaden substrate specificity toward ketoses, or screening heterologous transporters with relaxed substrate specificity. For vacuolar storage, tonoplast sugar transporter (TST) is engineered to acquire specific D-allulose recognition capacity, enabling active vacuolar transport while preserving native sucrose transport function, achieving parallel sucrose and D-allulose accumulation [77,78].

### 4.3 Pathway design for D-tagatose in a plant chassis

The design described above is specific to D-allulose, because hydrolysis of stored sucrose in sink tissue supplies D-fructose, the direct substrate of DPEase. A route to D-tagatose cannot be obtained by transferring the canonical enzymology. L-arabinose isomerase requires free D-galactose, and sink parenchyma does not maintain a high-flux free galactose pool: galactose is channelled through UDP-galactose into cell-wall polysaccharides and galactolipids, and free galactose accumulation is growth-inhibitory in many species. Engineering a galactose supply of sufficient magnitude would therefore compete directly with cell-wall biosynthesis and is unlikely to be compatible with normal sink development. This asymmetry, rather than any difference in the maturity of the enzyme toolkit, is the reason that D-tagatose requires a distinct compartmentalised design.

A phosphorylated route is far better matched to the amyloplast environment. *In vitro* and whole-cell cascades convert D-fructose 6-phosphate to D-tagatose 6-phosphate through a fructose-6-phosphate 4-epimerase, followed by irreversible dephosphorylation catalyzed by a tagatose-6-phosphate phosphatase [12,46]. The amyloplast stroma already carries a hexose-phosphate pool connected to starch synthesis, so the sucrose import and invertase steps proposed for D-allulose (Fig. 3b) can be used unchanged, whereas the phosphorylation step is route-specific: here the fructose released by invertase must itself be phosphorylated, by a fructokinase or by a hexokinase with fructose activity, to D-fructose 6-phosphate, while in the D-allulose route the same fructose must be spared. The catalytic module is then exchanged (Fig. 3c), substituting the epimerase-phosphatase pair for DPEase. Because the dephosphorylation step is thermodynamically irreversible, this design removes the equilibrium ceiling that constrains isomerase-based D-tagatose production, in the same way that the phosphorylation-dephosphorylation cascade does for D-allulose *in vitro*.

Three requirements distinguish this route from the D-allulose design and must be tested explicitly. First, partitioning of carbon between the engineered branch and starch synthesis has to be quantified, since diverting flux from the amyloplast's native carbon deposit may reduce sink strength. Second, phosphate balance becomes a design variable rather than a background condition, the phosphatase releases inorganic phosphate into a compartment in which the Pi/triose-phosphate ratio regulates ADP-glucose pyrophosphorylase and hence starch synthesis itself [76], so an uncontrolled Pi release could perturb the very metabolism that supplies the substrate. Third, export and vacuolar storage face the same transporter constraints described above for D-allulose, with the additional requirement that D-tagatose is a documented antimicrobial and defence-eliciting molecule [[27], [28], [29]], so its accumulation in living tissue may itself alter plant physiology. No crop-level D-tagatose titer has been reported, and this route is therefore presented as a testable design rather than a demonstrated capability.

The single-plasmid system scaffold is shared across all three host species, with only promoter selection and enzyme adaptation tailored to each chassis (Supplementary Table S1) [[79], [80], [81], [82], [83], [84], [85], [86], [87], [88], [89]]. *S. bicolor* serves as the primary validation platform, *Saccharum* spp. hybrids as the scaled production chassis [64,65] with expression driven by the mature stem-specific promoter *TST2b-1A* [90], and *B. vulgaris* subsp. *vulgaris* as the temperate complementary chassis, with a prerequisite assessment of amyloplast abundance/activity during the sugar storage phase [91] and potential introduction of plastid division protein FtsZ to maintain reaction compartment capacity [92].

### 4.4 From “agricultural extraction” to “decentralized” manufacturing

Plant biofactories mark a fundamental reshaping of the sugar industry logic. The Sugar 1.0 model passively obtains plant-synthesized sucrose with low added value, while the Sugar 4.0 era reprograms crop metabolic networks to actively guide plant cells to synthesize scarce D-allulose or D-tagatose, achieving a leap from “growing what is available” to “growing what is needed”.

A second consequence concerns where the conversion takes place. Traditional microbial fermentation relies on energy-intensive, capital-heavy fermenters [93], whereas a plant biorefinery would in principle relocate the reaction compartment into the vacuoles and plastids of a growing crop, so that harvesters collect a juice already enriched in the target sugar rather than a raw material for subsequent conversion. Such a model would avoid fermenter depreciation and the associated electricity demand, and would couple the added value of synthetic biology to the low marginal cost of agricultural cultivation. It is important to state the limits of this argument. Field production does not remove extraction, purification, quality control or regulatory oversight, and it introduces its own energy, land, water and fertiliser inventory. Carbon fixed by the crop constitutes a net climate benefit only to the extent that it is retained in the product or in soil rather than returned to the atmosphere through residue decomposition or processing. Any claim of low-carbon or negative-carbon manufacture must therefore rest on a comparative life-cycle assessment with an explicit system boundary, which is the subject of the following section.

## 5 Industry prospects and challenges

Translating laboratory advances into widely available products will depend primarily on improving production efficiency and reducing resource inputs. Closing the price gap between healthy sugars and sucrose therefore requires progress in both the underlying metabolic design and the manufacturing process. The healthy sugar industry has formed two converging technological evolution logics: industrial lean practices to enhance molecular conversion efficiency, and agricultural intensification to reduce foundational inputs, which together form the basis for industry-wide cost reconstruction in the era Sugar 4.0.

### 5.1 Dual-track driven cost reduction logic and global dividends

Commercial substitution of sucrose requires a substantial reduction in the delivered cost of healthy sugars, and two partly independent routes are being pursued. On the industrial side, thermodynamically driven cascades replace purified fructose or galactose with starch or maltodextrin and raise single-pass yield well above the epimerization ceiling, which reduces the chromatographic separation duty that dominates cost in equilibrium-limited processes [11,12,42]. On the agricultural side, a plant chassis would move carbon fixation and the first conversion steps into field-grown biomass. The magnitude of any resulting cost advantage cannot yet be estimated. No peer-reviewed unit-cost figures are available for either the cell-free or the plant route (Table 2), and the prerequisites for a plant-chassis advantage, an in planta titer high enough to justify a dedicated extraction train, no agronomic yield penalty, and a separation process able to resolve D-allulose or D-tagatose from the sucrose, glucose and fructose present in the same juice, are at present all unmet. Claims that plant biofactories would substantially reduce production cost relative to industrial enzymatic routes should therefore be read as a target conditional on these prerequisites, not as a demonstrated result. Regulatory developments are similarly uneven across jurisdictions rather than uniformly permissive, and are examined separately below.

### 5.2 Technological bottlenecks and public acceptance

Despite this potential, the popularization of healthy sugars still faces multiple severe challenges. First, high healthy sugar accumulation in sugar crops involves possible interference with endogenous sugar signalling. Healthy sugars might interact with the hexokinase-dependent sugar-sensing system that reports carbon status in plant cells [68] and thereby perturb source-to-sink transport. This has not been tested for D-allulose or D-tagatose and is raised here as a risk to be evaluated rather than an established effect. High-concentration solute accumulation can also drastically alter intracellular osmotic pressure, potentially leading to stunted growth or reduced stress resistance if vacuolar directional isolation is not achieved. Therefore, constructing an “undisturbed growth” independent synthesis chamber is central to achieving a biomass and yield win-win. Second, healthy sugars in crop juice coexist with high concentrations of sucrose, fructose, glucose, cellulose and ash. The extreme physical similarity between healthy sugars and their isomers makes low-cost, high-purity large-scale purification a significant challenge for existing SMB chromatography technologies [42]. Backend separation cost offsets may negate front-end agricultural cost advantages if the “precise isomer capture” bottleneck is not overcome.

Current research primarily focuses on D-allulose and D-tagatose, which represents only a small part of the accessible structural space. A large number of natural sugar isomers with untapped health potential exist in nature [44], but their low natural abundance makes it difficult to obtain sufficient samples for clinical evaluation [43]. Leveraging high-throughput screening platforms combined with AI metabolic models to accurately predict sugar molecules’ physiological functions is crucial for transforming Sugar 4.0 from generalized alternatives to precision nutrition.

Finally, plant biomanufacturing depends on efficient genetic transformation and, for multi-locus edits, on CRISPR/Cas genome editing [94]. In markets with stringent regulation of genetically engineered organisms, the choice of transformation strategy determines not only technical feasibility but also the regulatory pathway and the accessible market, as set out in the section on regulation and biosafety below.

### 5.3 Comparative life-cycle and techno-economic considerations

Comparisons between manufacturing platforms are meaningful only when the functional unit and the system boundary are stated. For healthy sugars the appropriate functional unit is 1 kg of food-grade, crystallised product delivered at specification, and the boundary must extend from feedstock production through conversion, separation, crystallisation and waste treatment. No harmonized life-cycle assessment covering industrial enzymatic, cell-free and plant-chassis routes has been published for either D-allulose or D-tagatose. Rather than presenting figures that do not exist, Table 3 sets out the inventory items that differentiate the three platforms, the direction of the expected effect, and the data that would be required to close each gap.

**Table 3:** Comparative life-cycle and techno-economic framework for healthy-sugar manufacturing platforms.

Inventory item	Industrial enzymatic (immobilized epimerase)	Cell-free enzymatic cascade	Plant biofactory (proposed)	Data required to close the gap	References
Feedstock	Purified D-fructose or D-galactose; high-value, derived from starch or lactose processing	Starch or maltodextrin; substantially cheaper per carbon unit	Atmospheric CO_2_ fixed by the crop; no purchased sugar feedstock	Mass balance per kg product for each route, including feedstock upstream burdens	[11,51]
Energy input	Reaction heating, pumping and, dominantly, chromatographic separation and evaporation	Reaction heating plus enzyme production; separation load reduced in proportion to higher yield	Field operations, irrigation, harvest and transport, plus juice extraction and evaporation	Cumulative energy demand per kg product under a common functional unit	[11,42]
Biocatalyst supply and replacement	Immobilized enzyme with defined operational lifetime; carrier replacement recurring	Five or more recombinant enzymes; half-life and reuse count determine replacement frequency	Enzymes expressed in planta and renewed each growing season; no separate catalyst manufacture	Enzyme production inventory per kg product; measured operational half-life at scale	[12,[35], [36], [37],^40^]
Separation and purification	SMB chromatography to resolve product from residual isomeric substrate; dominant cost term	Reduced but not eliminated; irreversible step raises yield and lowers separation duty	Product co-accumulates with sucrose, glucose and fructose in juice; separation burden potentially the highest of the three	Pilot separation trials on authentic engineered juice; energy and yield of each separation stage	[42]
Land, water and fertiliser	Indirect, through the feedstock supply chain	Indirect, through the starch supply chain	Direct and substantial; competes with the existing sucrose supply chain for the same land	Allocation between sucrose and healthy sugar as co-products of a single crop	[62]
Carbon balance	Net emitter; determined by process energy and feedstock upstream burden	Net emitter; CO_2_ as a carbon source does not by itself confer a credit	Fixation credit conditional on carbon retention in product and soil, and on residue management	Full carbon accounting including residue fate, processing emissions and land-use change	[50,51]
Capital intensity	Established; reactors, columns and crystallisers amortised over commercial volumes	High per unit output at current scale; enzyme production facilities required	Low incremental capital where existing mills are used, but new separation trains required	Capital and operating cost breakdown at a defined production scale	[52]
Current evidence status	Commercial operation; cost data proprietary	Laboratory demonstration; no published techno-economic analysis	Design proposal; no crop-level data of any kind	Published, peer-reviewed LCA and techno-economic analysis for all three routes	[11,12,31]; this review

Note: Entries state the direction and dominance of each inventory term rather than quantified values, because no harmonized life-cycle assessment of these platforms has been published for D-allulose or D-tagatose. References are given on each row. The direction stated in each cell is the present authors' assessment of the cited evidence rather than a value taken from a published inventory.

Three observations follow. First, cell-free systems avoid the carbon and energy costs of biomass formation but transfer that burden to recombinant enzyme production, cofactor or polyphosphate supply and periodic catalyst replacement. The reported biocatalyst half-lives and reuse counts (Table 2) are therefore the dominant term in any life-cycle inventory for these routes. Second, for a plant route the controlling terms are crop yield per hectare, the fraction of fixed carbon diverted into the target molecule, and the energy required to purify it from a complex juice. The agricultural inventory is comparatively well characterized, whereas the diversion fraction and the separation energy are unknown. Third, in all three platforms the resolution of a ketohexose from its structurally similar isomers is the step most likely to dominate cost and cumulative energy demand [42], so improvements in front-end conversion deliver benefit largely through the reduced separation load they create. A plant-chassis route would face this constraint in its most severe form, since the target sugar would accumulate alongside sucrose at far higher concentration.

### 5.4 Regulatory frameworks, environmental risk, and public acceptance

Two distinct regulatory pathways govern a plant-derived healthy sugar, and conflating them has been a persistent source of over-optimism. The first concerns the ingredient itself, and is already partly resolved. The second concerns the genetically engineered crop that would produce it, and is not. Approval of D-allulose or D-tagatose as a food ingredient in a given market says nothing about whether a transgenic sugarcane or sugar beet accumulating that sugar could be cultivated or its product marketed there.

At ingredient level, the position differs markedly between jurisdictions (Supplementary Table S2). In the United States, D-allulose is covered by multiple GRAS notifications to which the FDA has responded without objection, and since 2019 the agency has exercised enforcement discretion permitting its exclusion from the “Total Sugars” and “Added Sugars” declarations, with an energy factor of 0.4 kcal g^−1^ [45]. D-tagatose received a no-questions letter in 2001 with an energy factor of 1.5 kcal g^−1^. In the European Union the two molecules diverge sharply: D-tagatose has been authorised as a novel food since 2005 and appears in the Union list [95], whereas the EFSA Panel on Nutrition, Novel Foods and Food Allergens concluded in June 2025 that the safety of D-allulose could not be established because the applicant did not supply the requested data on identity, production process, use levels, genotoxicity and human studies [96]. D-allulose therefore remains unauthorised in the EU. In China, D-tagatose was approved as a new food raw material in 2014 [97], and D-allulose in July 2025 under Announcement No. 4 of 2025, covering both a fermentation route using *Escherichia coli* AS10 and an enzymatic route from D-fructose. This was the first new food raw material approved in China whose production process involves a genetically modified microorganism [98]. Permitted use levels are governed by GB 2760-2024 [99]. The picture is therefore one of asymmetric and still-moving regulation rather than uniform liberalisation, and market projections should be constructed jurisdiction by jurisdiction.

Environmental risk assessment is the element least addressed in the existing literature, and it differs substantially between the three chassis proposed here. Under the framework used for genetically modified plants [100], the principal concerns are transgene flow to sexually compatible wild or weedy relatives, persistence and invasiveness, and effects on non-target organisms. *Saccharum* spp. hybrids present the lowest gene-flow risk: commercial cane is propagated vegetatively, flowering is absent or suppressed in most production environments, and the aneuploid interspecific hybrids have poor fertility, although wild *S. spontaneum* populations occur across tropical Asia and volunteer ratoon persistence would require management. *B. vulgaris* subsp. *vulgaris* presents the highest risk in European production areas, being cross-compatible with wild sea beet (*B. vulgaris* subsp. *maritima*) along Atlantic and Mediterranean coasts and with weed beet populations already established within beet fields. Bolting control and harvest before flowering would be mandatory rather than advisable. *S. bicolor* is cross-compatible with johnsongrass (*S. halepense*) and with wild and weedy sorghum races in Africa and parts of the Americas, which argues for its use principally as a contained experimental platform, consistent with the role assigned to it here, rather than as an open-field production chassis in those regions.

Effects on non-target organisms require specific attention because the engineered product is not metabolically inert. D-tagatose has documented antimicrobial activity against oral species and inhibitory activity against oomycete pathogens [27,29], and both sugars are poorly metabolised by many microorganisms. Tissue accumulating them at high concentration would therefore be expected to alter the phyllosphere and rhizosphere microbiota and the decomposition of crop residues returned to the field. The same property that confers the sweet-immunity benefit described earlier is thus the property requiring ecological evaluation, and it cannot be assumed benign because the molecule is a food ingredient. Confinement strategies should be designed in from the outset. Plastid transformation largely prevents pollen-mediated transgene transmission and is mechanistically compatible with the amyloplast-targeted architecture proposed here. Other methods include male sterility, spatial isolation, and harvest before flowering.

Finally, even a shift from process-based to product-based regulation would not remove the practical constraints on industrial translation. Variety registration, food-safety assessment of the engineered crop as distinct from the purified ingredient, labeling obligations that differ between markets, and consumer acceptance of foods derived from genetically engineered crops all remain in force. A healthy sugar produced by microbial fermentation and one produced by a transgenic crop are chemically identical but face different market access, and this asymmetry, rather than the underlying science, may prove decisive for which route reaches consumers first.

### 5.5 Future directions

The strongest human evidence for both molecules concerns acute postprandial glycaemic and insulinaemic responses [13,14,23]. What is largely absent is long-term evidence: randomised controlled trials of sufficient duration to establish whether sustained substitution of sucrose produces durable changes in glycaemic control, body composition or cardiovascular risk markers. Two further questions require systematic study before population-scale recommendations are appropriate. The first is gastrointestinal tolerance, since both sugars are incompletely absorbed and reach the colon, where fermentation generates the butyrate that constitutes part of their claimed benefit [24]. The dose-tolerance relationship determines the practical ceiling on inclusion levels in foods. The second is the interaction with the gut microbiota over months rather than days, which is the timescale on which microbiome-mediated effects would manifest.

The cost trajectory will be determined by three variables: the price of the carbon feedstock, the achievable single-pass yield, and the separation duty implied by that yield. The transition from purified fructose or galactose to starch and maltodextrin addresses the first, and thermodynamically driven cascades address the second and, indirectly, the third. Rapid capacity expansion, particularly in China following the 2025 ingredient approval, should compress prices through conventional scale and competition effects independently of further technological change. The narrower question relevant here is at what point, if ever, a plant-chassis route becomes competitive with an optimized cell-free or fermentation process. The answer depends on the prerequisites set out in Table 3, and above all on whether an engineered crop can accumulate the target sugar at a concentration sufficient to justify a dedicated separation train alongside existing sucrose recovery. Until an in planta titer is measured, plant biofactories remain a research direction with a plausible economic rationale rather than a costed alternative.

Consumer response will not follow automatically from regulatory approval or favourable cost. The labeling asymmetry described above means that the same molecule carries different consumer-facing information in different markets, complicating formulation for international brands. Acceptance of foods produced using synthetic biology is uneven and depends strongly on how the production organism is described. The Chinese approval of a D-allulose process involving a genetically modified microorganism is a significant precedent, but a genetically engineered crop grown in open fields is a different proposition from a contained fermentation. Finally, the sensory advantage of these sugars over high-intensity sweeteners is realized only in applications where bulk, moisture retention and Maillard browning matter. Identifying and prioritising those applications is likely to determine early commercial success more than any further gain in conversion efficiency. Coordinated development of the health evidence base, the manufacturing platform and the consumer proposition, rather than sequential optimization of each, is the condition under which Sugar 4.0 becomes an industrial reality rather than a technological possibility.

## 6 Conclusions

The history of human sugar use is undergoing a profound transition from “extraction” (Sugar 1.0, sucrose) [7,8], “hydrolysis” (Sugar 2.0, high fructose syrup) [9], and “chemical synthesis” (Sugar 3.0, aspartame) [10] to “precision biomanufacturing” (Sugar 4.0). The core driving force behind this transformation is our mastery of life molecule reprogramming through synthetic biology. This paper reviews the two major technological pillars supporting this transformation, which together build the bridge for healthy sugars to move from the laboratory to the dining table.

This transformation is supported by two complementary technological pillars, cell-free pathway design and plant chassis engineering. Cell-free systems introduce a metabolic ratchet by coupling artificial phosphorylation cascades to irreversible dephosphorylation [11,12,47], overcoming the thermodynamic ceiling of conventional enzymatic isomerization and raising documented yields from below 40% to between roughly 79% and 88% at laboratory scale, with the atom economy needed to convert expensive reagents into affordable industrial products. Plant biofactories, by contrast, are proposed here as a design rather than a demonstrated platform: compartmentalised engineering of sugarcane, sugar beet and sorghum could in principle turn established crops into solar-powered autotrophic reactors, with a possible additional benefit from the sweet-immunity mechanism, but the cost and carbon advantages of this route remain conditional on prerequisites that have not yet been tested (Table 2, Table 3).

In the future, the pace at which these technologies reach consumers will be set jointly by advances in gene editing and transformation, by the accumulation of long-term human evidence, and by the divergent regulatory trajectories described above. If those conditions are met, D-allulose and D-tagatose could move beyond niche dietary products to become mainstream ingredients of the food industry, combining a sugar-like sensory experience with a markedly different metabolic fate, and offering a route by which reduced glycaemic burden, microbiota modulation and reduced cariogenicity can be delivered through everyday foods rather than through restriction. Realising that outcome will depend less on any single technological breakthrough than on the systematic closing of the evidence gaps identified in this review: crop-level titer, comparative life-cycle and techno-economic data, long-term clinical evidence, and jurisdiction-specific regulatory clearance for both the ingredient and, where relevant, the crop that produces it.

## Supplementary files

These files could be found online: https://github.com/zhangzihao252525/healthy-sugar/tree/main.

## Author contributions

Y.Q. and F.C. conceived the project. Z.Z., J.L., J. Z. and Q.W. collected the data and drafted the manuscript. Z.Z., J.L. and J. Z. prepared figures. Y.Q. and F.C. critically revised the manuscript. All authors read and approved the final manuscript.

## Data availability statement

Data sharing not applicable to this article as no datasets were generated or analyzed during the current study.

## Funding

This work was funded by the Innovation Team of National Tropical Agricultural Science Center (CATASCXTD202402), Project of State Key Laboratory of Tropical Crop Breeding (NKLTCBCXTD24 and NKLTCBCXTD38), Central Public-interest Scientific Institution Basal Research Fund (1630052024003, 1630052025013 and 1630052025021), and China Agriculture Research System of MOF and MARA (CARS-17).

## Declaration of competing interests

The authors declare that they have no known competing financial interests or personal relationships that could have appeared to influence the work reported in this paper.

## Figures and Tables

**figs1:**