# Supplementary material for: Sugar 1.0 to 4.0: From conventional sugar production to precision biomanufacturing of healthy sugars
Source: Biodes Res. 2026 Oct 1;8(4):100125. doi: 10.1016/j.bidere.2026.100125 (PMC13717879; doi:10.1016/j.bidere.2026.100125)
Supplement: Supplementary file 1 [file mmc1.docx]

**Supplementary Materials**

**Sugar 1.0 to 4.0: From conventional sugar production to precision biomanufacturing of healthy sugars**

Zihao Zhang, Junlin Li, Jiahao Zhang, Qibin Wu, Fei Chen*, Youxiong Que*

*Corresponding authors: Fei Chen (feichen@hainanu.edu.cn) and Youxiong Que (queyouxiong@126.com)

**Contents**

**Supplementary Fig. S1**  Natural enzyme sources and engineering chassis for healthy sugar production.

**Supplementary Table S1**  Tissue-specific promoters for heterologous expression in sugar crop chassis.

**Supplementary Table S2**  Comparison of regulatory status and frameworks relevant to healthy sugars and to plant-chassis production across major markets.

Supplementary Fig. S1 Natural enzyme sources and engineering chassis for healthy sugar production. The left panel groups the seven genera by their role in the strategy described in the main text rather than by phylogeny: bacteria as donors of catalytic parts, and angiosperms as the sites of natural occurrence and as recipient crop chassis. Bracket depths carry no evolutionary meaning and no common ancestry is implied between the two groups. *Pseudomonas* is the source of D-psicose 3-epimerase (DPEase), exemplified by *P. cichorii*, and Geobacillus is shown as a representative of the extremophilic thermophiles from which L-arabinose isomerase (L-AI) is obtained (Li et al., 2019). Among the eudicots, D-tagatose occurs in the gum exudate of *Sterculia setigera* Delile (Hirst et al., 1949) and D-allulose has been measured at trace levels in dried figs from Ficus carica L. among other plant-derived foods (Oshima et al., 2006); *Beta*, *Saccharum* and *Sorghum* are the three recipient chassis of the climate-adaptive manufacturing matrix described in the main text — *B. vulgaris* subsp. *vulgaris* (temperate), *Saccharum* spp. hybrids (tropical) and *S. bicolor* (model species). In the table, “described species” and “assembled genomes” are approximate figures compiled from public taxonomic and sequence databases; they are indicative only, are not used in any argument in the main text, and change with database releases. “Role in the engineering strategy” is the function assigned to each genus in this review, and “sugar products” gives the healthy sugar associated with the genus, either as a natural product or, for the three chassis, as the intended engineered product derived from stored sucrose. Em dashes indicate that the entry does not apply. Organisms are drawn schematically and are not to scale; bacterial cells and plants are shown at different magnifications.

**Supplementary Table S1 Tissue-specific promoters for heterologous expression in sugar crop chassis.** Promoters are grouped by the sink organ in which activity has been reported. Reported activity refers to the expression pattern described in the cited study and has not been re-evaluated here; promoter activity must be validated in each recipient species before use. Promoters differ in the cell type in which activity has been reported. Only those active in storage parenchyma — TST2b-1A/C in sugarcane stem and the tuber and storage-root promoters StPat, StGBSS, MeGBSSI and SRD1 — are directly compatible with the amyloplast-targeted design described in the main text, and TST2b-1A is the element selected there. The remaining entries fall into two groups: promoters whose reported activity is associated with lignifying or vascular tissue (SHDIR16, SHOMT, OsCesA7, OsMYB103L, SbSUT1, EgNAC141 and EgCAD2), and organ-preferential promoters for which the cited studies do not resolve the active cell type (SbTALE03/04, ZmGLU1, NtRB7 and AtRSK1). Both groups would require validation, and where necessary redirection, before use. The granule-bound starch synthase promoters StGBSS and MeGBSSI are of particular interest because the native gene product is itself amyloplast-localised.

| **Plant species** | **Promoter name** | **Tissue specificity** | **Applicable chassis** | **References** |
| --- | --- | --- | --- | --- |
| *Saccharum* spp. | *SHDIR16* | Stem, secondary cell wall or vascular tissue | *Saccharum* spp. / *Sorghum bicolor* | (Damaj et al., 2010) |
| *Saccharum* spp. | *SHOMT* | Stem, secondary cell wall or vascular tissue | *Saccharum* spp. / *Sorghum bicolor* | (Damaj et al., 2010) |
| *Saccharum* spp. | *TST2b-1A/C* | Stem, storage parenchyma | *Saccharum* spp. / *Sorghum bicolor* | (Wang et al., 2021) |
| *Oryza sativa* | *OsCesA7* | Stem, secondary cell wall or vascular tissue | *Saccharum* spp. / *Sorghum bicolor* | (Noda et al., 2015) |
| *Oryza sativa* | *OsMYB103L* | Stem, secondary cell wall or vascular tissue | *Saccharum* spp. / *Sorghum bicolor* | (Yang et al., 2014) |
| *Sorghum bicolor* | *SbTALE03/04* | Stem, cell type not resolved | *Saccharum* spp. / *Sorghum bicolor* | (Fu et al., 2025) |
| *Sorghum bicolor* | *SbSUT1* | Stem, phloem | *Saccharum* spp. / *Sorghum bicolor* | (Milne et al., 2013) |
| *Eucalyptus* spp. | *EgNAC141* | Stem, secondary cell wall or vascular tissue | *Saccharum* spp. / *Sorghum bicolor* | (Sun et al., 2021) |
| *Eucalyptus* spp. | *EgCAD2* | Stem, secondary cell wall or vascular tissue | *Saccharum* spp. / *Sorghum bicolor* | (Rahantamalala et al., 2010) |
| *Solanum tuberosum* | *StPat* | Root, storage parenchyma | *Beta vulgaris* | (Miroshnichenko et al., 2020) |
| *Solanum tuberosum* | *StGBSS* | Root, storage parenchyma | *Beta vulgaris* | (Miroshnichenko et al., 2020) |
| *Manihot esculenta* | *MeGBSSI* | Root, storage parenchyma | *Beta vulgaris* | (Koehorst-van Putten et al., 2012) |
| *Ipomoea batatas* | *SRD1* | Root, storage parenchyma | *Beta vulgaris* | (Noh et al., 2009) |
| *Zea mays* | *ZmGLU1* | Root, cell type not resolved | *Beta vulgaris* | (James et al., 2022) |
| *Nicotiana tabacum* | *NtRB7* | Root, cell type not resolved | *Beta vulgaris* | (James et al., 2022) |
| *Arabidopsis thaliana* | *AtRSK1* | Root, cell type not resolved | *Beta vulgaris* | (James et al., 2022) |

**Supplementary Table S2 Comparison of regulatory status and frameworks relevant to healthy sugars and to plant-chassis production across major markets.** Regulatory status is current at the time of writing and should be verified before commercial decisions are taken. —, no single primary source; the entry summarises several national frameworks. Full citations are given in the reference list of the main text.

| **Jurisdiction** | **D-allulose (ingredient status)** | **D-tagatose (ingredient status)** | **Framework applying to genetically engineered crops** | **Practical implication for a plant-chassis route** | **References** |
| --- | --- | --- | --- | --- | --- |
| United States | Multiple GRAS notifications with FDA no-objection responses; excluded from “Total Sugars” and “Added Sugars” declarations since 2019 under enforcement discretion; energy factor 0.4 kcal g⁻¹ | GRAS no-questions letter issued in 2001; energy factor 1.5 kcal g⁻¹ | USDA APHIS regulation of modified plants together with FDA premarket consultation for foods derived from them | Most permissive of the three markets for both ingredient and crop; a plant-derived product would still require its own safety evaluation | FDA, 2020 |
| European Union | Not authorised; EFSA concluded in June 2025 that safety could not be established because requested data were not supplied | Authorised as a novel food since 2005 and included in the Union list of novel foods | Directive 2001/18/EC and Regulation (EC) No 1829/2003; proposals on new genomic techniques under negotiation | Ingredient authorisation for D-allulose must be obtained before any plant-derived product; gene-flow risk for sugar beet is highest in this region | European Commission, 2017; EFSA GMO Panel, 2010; EFSA NDA Panel, 2025 |
| China | Approved as a new food raw material in July 2025 (Announcement No. 4 of 2025), covering a fermentation route and an enzymatic route; first such approval involving a genetically modified production organism; permitted use levels are governed by GB 2760-2024 | Approved as a new food raw material in 2014 (Announcement No. 10 of 2014) | Agricultural GMO safety certificate system, with separate evaluation guidelines for gene-edited plants | Ingredient pathway now open for both molecules; crop-level approval remains a separate and more demanding process | NHC, 2014; NHC, 2024; NHC, 2025 |
| Other markets (Japan, Republic of Korea, Australia and New Zealand) | In use in several Asian markets; status should be confirmed jurisdiction by jurisdiction before commercial planning | Reported as approved in several other markets, including the Republic of Korea, Australia and New Zealand, and evaluated by JECFA; the status in each market should be confirmed from the national authority before commercial planning. | National frameworks differ; several operate mandatory labeling of genetically engineered content | Labeling asymmetry between markets is likely to shape the commercial viability of a crop-derived product | — |
